# Differences in Parenting Behavior are Systematic Sources of the Non-shared Environment for Internalizing and Externalizing Problem Behavior

**DOI:** 10.1007/s10519-022-10125-8

**Published:** 2022-11-03

**Authors:** Amelie Nikstat, Rainer Riemann

**Affiliations:** Department of Psychology, Bielefeld University, Bielefeld, Germany

**Keywords:** Internalizing problem behavior, Externalizing problem behavior, Parenting, Gene-environment correlation, Monozygotic twin differences, Non-shared environment

## Abstract

Although there is evidence for non-shared environmental links between parenting and problem behavior, so far, age-, informant-, and parent-specific patterns for both internalizing and externalizing problem behaviors have not been examined within one study yet. Using the twin differences design, the present study aimed to test how maternal and paternal parenting systematically act as a source of non-shared environment for problem behavior across different age groups and informants. We examined 1327 monozygotic twin pairs and their parents drawn from three birth cohorts of the German TwinLife study. Our results revealed that particularly child-reported less positive and more negative parenting by both parents contribute significantly to the unique environmental variance of problem behavior, although we did not find a clear pattern across age groups. Our study underlines the necessity of controlling for genetic confounding to uncover the truly environmentally mediated (and thus environmentally influenceable) pathways between parenting and problem behavior. A practical implication could be that it may be useful to primarily consider the child’s perspective and focus on maternal as well as paternal parenting in interventions that address parenting to reduce problem behavior.

**Supplementary Information:**

The online version contains supplementary material available at 10.1007/s10519-022-10125-8.

## Introduction

Behavioral, emotional, and social problems in childhood and adolescence are generally grouped into two broader categories: Internalizing (INT) and externalizing (EXT) behavior problems (Achenbach 1966). INT is characterized by processes within the self and includes symptoms like anxiety, depression, and somatization, whereas EXT primarily occurs in interaction with the external world and manifests through symptoms such as aggression, antisocial behavior, and impulsivity. Both, INT and EXT are highly prevalent, associated with numerous unfavorable developmental outcomes and have a lasting impact on social relationships, educational and occupational success, health and quality of life (Hölling et al. 2014).

Among many different environmental factors, previous research identified parenting behavior as one of the most important influences on problem behavior (Carneiro et al. 2016). Parenting behavior is considered ‘a constellation of attitudes towards the child that are communicated to the child and that […] create an emotional climate in which the parent’s behaviors are expressed. These behaviors include both the specific, goal-directed behaviors through which parents perform their parental duties […] and non-goal-directed parental behaviors, such as gestures, changes in tone of voice, or the spontaneous expression of emotion’ (Darling and Steinberg 1993, S. 488), and remain relatively constant across different life situations (Hannigan et al. 2017).

In a recent literature review on associations between parenting and INT, Gorostiaga et al. (2019) showed that negative parenting behaviors (e. g., harsh control, negative communication) are positively associated with INT and conversely, positive parenting behaviors (e. g., parental warmth, monitoring) are inversely related to INT. Similarly, in a meta-analysis on associations between parenting and EXT, Pinquart (2017) observed moderate positive associations between negative parenting behaviors (particularly harsh control and psychological control) and EXT, though they found only weak associations with EXT for positive parenting behaviors (e. g., parental warmth, monitoring). Both studies emphasized that the conception of the relationship between parenting and problem behavior may be oversimplified and that numerous other influences could affect the relationship (e. g. genetic factors, socio-economic status, or the child’s age).

The well-established family stress model by Conger et al. (2010) describes a process in which parenting behavior acts as a mediator between economic hardship and pressure and problem behavior in children and adolescents via parental emotional and behavioral problems and parental conflict. Thus, above all, other aspects of the family environment influence the association between parenting and problem behavior. Since parents and their children are genetically related, the associations stated in the model may be due to common genetic variance. Consequently, if the association between parenting and problem behavior is assessed via traditional, non-genetically informative family designs such as the ‘between-family, one-child-per-family’ or the ‘within-family, two-children-per-family’ design (as usual in most family and child research), positive results could be confounded by genetic pathways (Jaffee and Price 2012). This mechanism is called gene-environment-correlation (rGE; Plomin et al. 1977) and occurs when exposure to environmental conditions depends on the genotype of an individual. Three different types of rGE have been described: *Passive* rGE refers to the fact that biological parents pass their genes on to their children, as well as providing the environmental conditions in which the child grows up, resulting in a correlation between a genetically influenced phenotype of the child and the family environment. In other words, the genetic makeup may be to some extent responsible for both, parenting and problem behavior of the child. *Evocative* rGE occurs when an individual’s genetically influenced phenotype evokes an environmental response, for example, a child who shows more problem behavior will evoke different responses from its parents than one who behaves inconspicuously. *Active rGE* happens when an individual has a heritable predisposition to a particular environmental exposure. It is a well-established finding by now that rGE plays an important role for almost all aspects of the social environment, including multiple aspects of parenting (Kendler and Baker 2007; Klahr and Burt 2014; Pike and Oliver 2015). For example, meta-analyses by Avinun and Knafo (2014) and Klahr and Burt (2014) showed that genetically influenced child behavior affects both positive and negative aspects of parenting, and Pike and Oliver (2015) concluded in their review that the association between parenting and EXT is mainly due to common genetic influences. Thus, since it can be assumed that rGE is an important mechanism in the association between parenting and problem behavior, it is essential to control for rGE to uncover truly environmentally mediated (and thus environmentally influenceable) pathways.

## Behavior genetic studies

Classical behavior genetic studies decompose phenotypic variance into variance due to genetic and environmental sources. Environmental sources can be divided into sources of the shared and the non-shared environment. Whereas shared environmental influences make children reared together more similar, non-shared environmental influences make children different from one another. Genetic, shared, and non-shared environmental influences together account for 100% of the variance (Plomin et al. 1990). According to univariate biometric models, a major part of the variance in problem behavior is accounted for by environmental influences, although heritability also plays an important role for both, INT and EXT (Nikstat and Riemann 2020). In addition to the variance decomposition, it is important to study which specific influences are responsible for the corresponding environmental variance components. Although recent work underlines the importance of shared environmental influences for psychopathology in childhood and adolescence (Burt 2009, 2014), across all studies on INT and EXT, non-shared environmental influences emerged as the most important environmental variance component. Non-shared environmental effects are often even larger than genetic influences, especially for INT, but are much harder to identify compared to shared environmental influences, as they mostly occur unsystematically, and are therefore difficult to measure and investigate in a systematic way (Turkheimer and Waldron 2000). Accordingly, hardly any actual systematic influences of the non-shared environment on problem behavior have been identified so far, and actual measured environmental differences tend to explain only a small part of the phenotypic variance between siblings, although in sum they account for a large part of the variance in behavioral genetic studies (Turkheimer and Waldron 2000).

### Twin differences method

The twin differences method (Jinks and Fulker 1970; Pike et al. 1996) provides a unique opportunity to examine the role of non-shared environmental influences on children’s and adolescents’ traits while controlling for genetic and shared environmental influences. The method allows for testing whether different environmental experiences (e. g., different parenting) are systematically responsible for trait differences (e. g., in problem behavior) between monozygotic twins (MZ) raised together. MZ are the same age and sex, share 100% of their genes and if raised together, they also share 100% of their family environment, meaning that any differences between them are due to non-shared environmental effects. In other words, the twin differences method allows to rule out the possibility that (1) age and sex differences between the siblings are responsible for different parental treatment, (2) other risk factors which vary between -but not within- families (e. g., low socioeconomic status) account for the association between parenting and problem behavior, (3) common genetic factors explain both parenting and child behavior (*passive* rGE), and that (4) genetically influenced differences between children cause different parental treatments (*evocative* rGE). Thus, if differences in parental treatment cause one twin to develop more behavioral problems than the other, that would be a direct measure of systematic non-shared environmental influence.

Parenting behavior often differs significantly between children in the same family (Cummings et al. 2000), and could therefore be a contributor to the non-shared environmental variance found in behavior genetic studies. Up until now, a few studies examined the relationship between parenting and problem behavior in a twin differences design (Asbury et al. 2003; Caspi et al. 2004; Chen et al. 2016; Deater‐Deckard et al. 2001; Guimond et al. 2016; Pike et al. 1996; Viding et al. 2009; Waller et al. 2018). Although these studies vary considerably in terms of methodology, there is a pattern that runs through the results: All studies found positive associations between negative parenting and problem behavior, whereas fewer studies found negative associations between positive parenting and problem behavior, and these mostly for INT. In other words, the twin who experiences more negative parenting also shows more INT and EXT compared to their co-twin, but the twin who experiences more positive parenting shows less INT, but not less EXT. Although the correlations found in the twin differences design tend to be rather low, this research shows that the relationship between parenting and problem behavior is partly environmentally mediated. However, much remains unclear, as so far age-, informant-, and parent-specific patterns for both internalizing and externalizing problem behaviors have not been examined within one study.

Previous studies differ widely regarding the examined age range, and mostly did not control for age effects. Two studies which considered age as a moderator variable did not find any associations between age and twin differences in problem behavior and parenting (Chen et al. 2016; Waller et al. 2018). But, if considered as moderator variable, only mean differences can be examined but not structural differences in the relationship between twin differences in parenting and problem behavior in different age groups. A systematic exploration of different age groups has yet to be conducted. Nevertheless, there is evidence that rGE increases by age, perhaps because older children become more autonomous and seek out social environments which match their genes (Vitaro et al. 2009). As a result, MZ twin differences would decrease with age, and this variance restriction would lead to lower correlations in older children, which underlines the importance of examining if different correlation patterns emerge in different age groups.

Furthermore, the consideration of subjective experiences is essential for the identification of non-shared environmental effects, because even shared environmental experiences can have non-shared effects, depending on how they are perceived (Turkheimer and Waldron 2000). For example, parental reports of parenting show fewer differences in the treatment of children than the reports of the twins themselves (Pike et al. 1996), which might be due to the fact that parent-reported differences are based on the ratings of the same informant (e. g., the mother), while differences in child reports are based on the ratings of different informants (both twins). Therefore, the parenting may be actually the same, but the twins experience it differently, leading to lower twin correlations and higher non-shared environmental effects. Reversely, the rating of both twins by a single informant could lead to higher twin correlations and lower non-shared environmental effects because of expectations that MZ twins should be treated or behave similarly (Vitaro et al. 2009). Additionally, while few studies with adolescents used child-reports, there are no twin differences studies that used child-reports in samples of younger children, although children’s perception of parenting shows considerable validity (Pike and Oliver 2015). As far as we know, with one exception (Pike et al. 1996), no study considered parental reports and child-reports about both parents in a twin differences design yet. Moreover, since in most families the mother is the primary caregiver, it is of particular interest to investigate whether differences in maternal parenting have a greater impact on twin differences than differences in paternal parenting. Although mothers still tend to spend more time with their children (Craig and Powell 2012), the father's role in parenting has become much more important over time (Lamb 2010) and in genetically non-informative designs paternal parenting shows similar associations to problem behavior as maternal parenting (Pinquart 2017). However, most previous genetically informed research used maternal reports, and the examination of the father’s role in the parenting environment is still underrepresented (Pike and Oliver 2015).

To our knowledge, maternal and paternal differences in parenting and their associations with differences in problem behavior have only been explicitly examined by Pike et al. (1996). They found a similar pattern of associations with differences in problem behavior for differences in paternal as well as in maternal parenting, with the exception that on average, the associations between differences in paternal negative parenting and twin differences in EXT were slightly higher than associations for differences in maternal negative parenting. All in all, the few findings on differences in maternal and paternal parenting indicate that fathers have no less, and maybe in detail even a higher influence than mothers. Therefore, genetically informed studies should examine the role of paternal parenting in addition to the role of maternal parenting in the development of problem behavior. Finally, Asbury et al. (2003) found stronger associations of twin differences in families with lower socioeconomic status (SES) and greater family chaos. Therefore, a representative sample which covers the whole range of key socio-demographic variables is very important if one is interested in general mechanisms.

## Aim of the current study

To test whether and how parenting is systematically responsible for non-shared environmental effects, the present study will examine the extent to which differences in parenting behavior affect differences in the expression of INT and EXT in MZ twin pairs. Our study is the first to examine this in a representative German sample (see Ho et al. 2008, for effects of culture) and to cover a wide age range by considering different twin birth cohorts. We also consider possible rater effects, as well as parenting x SES interaction and pay particular attention to the role of paternal parenting behavior. Our focus thereby is on identifying those dimensions of parenting that are most relevant to problem behavior after controlling for genetic confounding and whether these differ across age groups. To control for the overlap between dimensions of parenting, we examine the prediction of all parenting differences on behavior problem differences simultaneously. In general, we hypothesize that equally for both parents differences in negative parenting would positively predict and differences in positive parenting would negatively predict MZ differences in problem behavior, although we expect these associations to be weaker than those of non-genetically controlled analyses. In addition, we expect within-rater associations to be more likely and more significant than between-rater associations. Regarding possible age effects, our work is rather exploratory, thus we do not hypothesize the extent to which increasing autonomy with age affects the unique environmental contribution of parenting to the variance of problem behavior.

## Method

### Sample

Our sample included 1327 MZ twin pairs and their parents (if available) from three separate birth cohorts (C; on average aged 5, 11, and 17, Supplement 1) of the first wave of data collection (2014/2015) from the TwinLife study of social inequalities (see Hahn et al. 2016). All data is publicly available (Diewald et al. 2021). The TwinLife sample is representative of the German population and covers a wide variety of important indicators of social inequality like educational status, occupational status and income (Lang and Kottwitz 2017). We included all MZ twin pairs (C05: 51.7% female, C11: 54.6% female, C17: 56.2% female) with complete data on INT and EXT available. Detailed information on household structure and missing analyses can be found in Supplement 2.

### Measures

#### Zygosity

In C05 and C11, zygosity was determined with the *Zygosity Questionnaire for Young Twins* (Goldsmith 1991); in C17, zygosity was measured with the *Self Report Zygosity Questionnaire* (Oniszczenko et al. 1993). In addition, DNA-based zygosity for *N* = 328 twin pairs was determined and used as a criterion for questionnaire validity. This results in correct MZ classification rates of 98% and 97% for self- and parent-reports, respectively (see Lenau et al. 2017 for more details).

#### INT and EXT

INT and EXT were represented by four of the five subscales of the German translation of the *Strengths & Difficulties Questionnaire* (SDQ; Goodman 1997). The SDQ was developed as a screening instrument for psychosocial problems in children and adolescents in population-based samples and is available in both a child-report and a parental report version (see http://www.sdqinfo.org for both versions). For details on item wording for different raters and age groups in TwinLife see Klatzka et al. (2022). Consistent with the recommendations of Goodman et al. (2010), we combined the subscales *emotional problems* and *peer problems* to represent INT, whereas the subscales *hyperactivity* and *conduct* reflect EXT. Each subscale consists of five items, which were rated on a three-point scale (0 = *not true*, 2 = *certainly true*). INT and EXT scales were created as the average of the corresponding item responses. For the younger children in C05, parental reports of the SDQ were assessed. Participants aged 10 or older received the child-report version, respectively the child-report version adapted for adults if they were over 17 years old. Configural measurement invariance between the child- and the parental report was recently shown by Rogge et al. (2018), therefore both versions are comparable on the phenotypic level. Consistent with commonly reported reliabilities for the SDQ, reliabilities (Cronbach’s α) were 0.68 for INT and 0.70 for EXT across all cohorts (for a detailed overview see Supplement 3). Descriptive statistics can be found in Supplement 1.

#### Parenting

Parenting style was surveyed with 13 items on five scales, all of which were adapted from the pairfam study (Huinink et al. 2011). A detailed description of all utilized items and scales can be found in Klatzka et al. (2022). The scale *warmth* includes four items indicating the degree of affirmative attention and care in parenting, the scale *monitoring* consists of two items indicating the degree to which parents are informed about their child’s social contacts and activities, whereas the remaining three scales represent more negative aspects of parenting behavior: The three items of the scale *psychological control* assess negative thoughts, feelings and behavior towards the child, the scale *negative communication* includes two items indicating the degree of negative parental behavior towards the child, and the two items of the scale *inconsistent parenting* indicate the degree of inconsistency in parenting behavior.

Parenting was assessed via child report as well as paternal and maternal self-report, and the items were kept strictly parallel for both versions. All items were answered on a 5-point scale (1 = *never*, 5 = *very often*), respectively on a 3-point scale (1 = *never*, 3 = *very often*) for the child report in the youngest cohort. All parenting scales were computed as the average of the corresponding item responses. Exploratory factor analyses showed that for both child- and parent-reports, the scales *warmth* and *monitoring* could be combined into the broader dimension of *positive parenting*, while the three remaining scales could be combined into the broader dimension of *negative parenting* (see Supplement 4). Descriptive statistics can be found in Supplement 1. Reliability estimates (Cronbach’s α, Supplement 3) for *positive parenting* were 0.75 for child reports and 0.79 for parental reports across both parents and all cohorts, and 0.71 and 0.72 for *negative parenting*, respectively. Correlations between child- and parent-reported parenting ranged between 0.11 and 0.39, correlations between mother- and father-reported parenting ranged between 0.18 and 0.68 (for details see Supplement 5).

#### SES

We used the International Socio-Economic Index of Occupational Status (ISEI; Ganzeboom et al. 1992) as indicator for SES. The ISEI is based on the individual’s occupation, income and education on a continuous hierarchical scale ranging from 12 to 90. The ISEI was surveyed separately for each parent. We set the ISEI to the maximum of the maternal and paternal value for each twin pair to represent family SES (for descriptive statistics see Supplement 1).

## Analyses and results

Data preparation and preliminary analyses were conducted in SPSS 27.0 (IBM Corp. 2020). We computed relative difference scores for INT and EXT and all parenting variables, specifying the first-born MZ twin within a pair as twin 1 and subtracting the scores of twin 2 (Turkheimer and Waldron 2000; Vitaro et al. 2009). All correlations and multiple regressions were conducted in R 4.1.2 (R Core Team 2021). Given its advantages for handling missing data, Full Information Maximum Likelihood estimation was used for all correlation and regression analyses.

### MZ twin correlations

First, we examined to which extent problem behavior and parenting were influenced by non-shared environmental effects by calculating MZ correlations (r_MZ_) for each construct (Table 1). The difference 1-r_MZ_ indicates non-shared environmental effects and measurement error, which cannot be separated here. For child-reported parenting, INT, and EXT, all MZ correlations were moderate, pointing to substantial non-shared environmental contributions to the variance. In contrast, for parent-reported parenting the considerably higher correlations led to substantially smaller non-shared environmental contributions. In other words, consistent with previous genetic research (Avinun and Knafo 2014; Klahr and Burt 2014), parents tended to rate their parenting behavior towards the twins as more similar than it was perceived by the twins.

**Table 1:** Twin correlations

	C05 (N = 426)			C11 (N = 412)			C17 (N = 489)		
	*r*	*CI − *	*CI* +	*r*	*CI − *	*CI* +	*r*	*CI − *	*CI* +
INT^a^	0.51	0.44	0.58	0.41	0.33	0.49	0.44	0.37	0.51
EXT^a^	0.51	0.43	0.57	0.33	0.24	0.41	0.40	0.32	0.47
CR mother positive parenting	0.61	0.55	0.67	0.37	0.28	0.45	0.60	0.54	0.65
CR mother negative parenting	0.38	0.30	0.46	0.38	0.29	0.46	0.53	0.47	0.59
CR father positive parenting	0.61	0.55	0.67	0.55	0.48	0.62	0.58	0.52	0.64
CR father negative parenting	0.42	0.34	0.50	0.42	0.33	0.49	0.58	0.52	0.63
PR mother positive parenting	0.88	0.86	0.90	0.90	0.89	0.92	0.89	0.87	0.91
PR mother negative parenting	0.86	0.83	0.88	0.90	0.88	0.92	0.90	0.89	0.92
PR father positive parenting	0.89	0.86	0.90	0.94	0.93	0.95	0.91	0.89	0.92
PR father negative parenting	0.89	0.87	0.91	0.87	0.84	0.89	0.91	0.89	0.92

*C* cohort, *CI* 95% confidence interval, *INT* internalizing, *EXT* externalizing, *PR* parental report, *CR* child report

All correlations are significant with *p* < 0.001

^a^For C05, INT & EXT were assessed via parental report

### Phenotypic correlations

To avoid grouping effects, the correlations of parenting, SES, and sex with problem behavior were calculated for one randomly chosen twin from each twin pair. Overall, we found the expected pattern with positive associations between negative parenting and problem behavior and negative associations between positive parenting and problem behavior. If the same informant rated both problem behavior and parenting, associations were mostly higher than relations between data from different raters. SES showed substantial negative associations with problem behavior only in the youngest cohort, although a weaker association with INT was found in C17. Sex differences were found for EXT in C05, with higher levels of EXT in boys, and for INT in C17, with higher levels of INT in girls. Table 2 shows all phenotypic correlations.

**Table 2:** Phenotypic correlations between parenting and INT/EXT (random twin)

	INT				EXT			
	*r*	*p*	*CI − *	*CI* +	*r*	*p*	*CI − *	*CI* +
C05 (N = 426)								
CR mother positive parenting	− 0.03	0.515	− 0.13	0.06	− 0.03	0.592	− 0.12	0.07
CR mother negative parenting	0.02	0.719	− 0.08	0.11	0.26	**0.000**	0.17	0.35
CR father positive parenting	− 0.10	**0.032**	− 0.20	− 0.01	− 0.03	0.508	− 0.13	0.06
CR father negative parenting	− 0.06	0.216	− 0.15	0.04	0.23	**0.000**	0.14	0.32
PR mother positive parenting	− 0.11	**0.025**	− 0.20	− 0.01	− 0.19	**0.000**	− 0.28	− 0.09
PR mother negative parenting	0.19	**0.000**	0.10	0.28	0.34	**0.000**	0.26	0.42
PR father positive parenting	− 0.10	**0.039**	− 0.19	− 0.01	− 0.15	**0.002**	− 0.24	− 0.06
PR father negative parenting	0.14	**0.005**	0.04	0.23	0.20	**0.000**	0.11	0.29
Sex twins^a^	− 0.02	0.754	− 0.11	0.08	− 0.13	**0.006**	− 0.22	− 0.04
SES	− 0.19	**0.000**	− 0.28	− 0.09	− 0.26	**0.000**	− 0.35	− 0.17
C11 (N = 412)								
CR mother positive parenting	− 0.09	0.086	− 0.18	0.01	− 0.18	**0.000**	− 0.27	− 0.08
CR mother negative parenting	0.21	**0.000**	0.11	0.30	0.24	**0.000**	0.14	0.33
CR father positive parenting	− 0.08	0.108	− 0.18	0.02	− 0.16	**0.001**	− 0.25	− 0.06
CR father negative parenting	0.13	**0.011**	0.03	0.22	0.23	**0.000**	0.13	0.32
PR mother positive parenting	− 0.04	0.379	− 0.14	0.05	0.06	0.217	− 0.04	0.16
PR mother negative parenting	0.09	0.062	− 0.01	0.19	0.18	**0.000**	0.09	0.27
PR father positive parenting	− 0.09	0.061	− 0.19	0.00	− 0.04	0.473	− 0.13	0.06
PR father negative parenting	0.07	0.179	− 0.03	0.16	0.12	**0.013**	0.03	0.22
Sex twins^a^	0.05	0.272	− 0.04	0.15	− 0.05	0.318	− 0.15	0.05
SES	− 0.02	0.716	− 0.11	0.08	0.02	0.640	− 0.07	0.12
C17 (N = 489)								
CR mother positive parenting	− 0.13	**0.003**	− 0.22	− 0.04	− 0.18	**0.000**	− 0.26	− 0.09
CR mother negative parenting	0.17	**0.000**	0.08	0.25	0.28	**0.000**	0.19	0.36
CR father positive parenting	− 0.13	**0.004**	− 0.22	− 0.04	− 0.07	0.111	− 0.16	0.02
CR father negative parenting	0.07	0.127	− 0.02	0.16	0.18	**0.000**	0.10	0.27
PR mother positive parenting	0.01	0.902	− 0.08	0.09	− 0.03	0.559	− 0.12	0.06
PR mother negative parenting	0.01	0.875	− 0.08	0.10	0.21	**0.000**	0.12	0.29
PR father positive parenting	− 0.02	0.741	− 0.10	0.07	0.06	0.228	− 0.03	0.14
PR father negative parenting	− 0.02	0.689	− 0.11	0.07	0.13	**0.004**	0.04	0.22
Sex twins^a^	0.30	**0.000**	0.22	0.38	− 0.05	0.238	− 0.14	0.04
SES	− 0.10	**0.028**	− 0.19	− 0.01	− 0.08	0.071	− 0.17	0.01

Bold indicates *p* < .05

*INT* internalizing, *EXT* externalizing, *C* cohort, *CI* 95% confidence interval, *PR* parental report, *CR* child report, *SES* socio-economic status

^a^1 = male, 2 = female

### Twin differences

As expected, compared to the phenotypic correlations, the correlations in the twin differences design were for the most part smaller and in several cases not statistically significant anymore when different raters judged parenting and problem behavior. In C05, the correlation pattern was ambiguous, as in two cases the association between positive parenting and INT was positive, and in one case the association between negative parenting and EXT was negative, suggesting that the twin for whom parents reported more INT was treated more positively, whereas the twin for whom parents reported more EXT was treated less negatively than their co-twin. Also, in C05 parents reported significantly fewer differences in EXT when their SES was lower, and fewer differences in INT for boys than for girls, whereas all other associations between SES as well as sex and differences in problem behavior remained non-significant across all cohorts.

With one exception only associations between child-reported parenting and child-reported problem behavior remained significant in C11 and C17. In line with the phenotypic correlations, the twin who reported more problem behavior also perceived to be treated more negatively and less positively by their parents than their co-twin. All genetically controlled correlations are found in Table 3. To test for structural differences between C11 and C17, we compare correlation coefficients with Fisher’s *z* (Supplement 6) and found significant differences for child reported maternal negative parenting for INT (*z* = 2.25, *p* = 0.024) and parent reported maternal negative parenting for EXT (*z* = -2.24, *p* = 0.025), therefore we decided to run the following analyses separately for the cohorts.

**Table 3:** Correlations between twin differences in parenting and twin differences in INT/EXT

	INT				EXT			
	*r*	*p*	*CI − *	*CI* +	*r*	*p*	*CI − *	*CI* +
C05 (N = 426)								
CR mother positive parenting	0.12	**0.013**	0.03	0.21	0.07	0.134	− 0.02	0.17
CR mother negative parenting	− 0.03	0.546	− 0.12	0.07	0.00	0.958	− 0.10	0.09
CR father positive parenting	0.07	0.176	− 0.03	0.16	− 0.05	0.316	− 0.14	0.05
CR father negative parenting	0.00	0.950	− 0.09	0.10	− 0.11	**0.020**	− 0.21	− 0.02
PR mother positive parenting	0.09	0.074	− 0.01	0.18	− 0.14	**0.005**	− 0.23	− 0.04
PR mother negative parenting	0.04	0.466	− 0.06	0.13	0.10	**0.048**	0.00	0.19
PR father positive parenting	0.10	**0.044**	0.00	0.19	0.04	0.426	− 0.06	0.13
PR father negative parenting	0.00	0.981	− 0.09	0.10	0.11	**0.021**	0.02	0.20
Sex twins^a^	− 0.10	**0.040**	− 0.19	− 0.01	0.00	0.987	− 0.09	0.10
SES	− 0.05	0.309	− 0.14	0.05	− 0.14	**0.005**	− 0.23	− 0.04
C11 (N = 412)								
CR mother positive parenting	− 0.11	**0.033**	− 0.20	− 0.01	− 0.14	**0.003**	− 0.24	− 0.05
CR mother negative parenting	0.19	**0.000**	0.09	0.28	0.16	**0.001**	0.06	0.25
CR father positive parenting	− 0.03	0.589	− 0.12	0.07	− 0.15	**0.003**	− 0.24	− 0.05
CR father negative parenting	0.17	**0.000**	0.08	0.26	0.20	**0.000**	0.10	0.29
PR mother positive parenting	0.05	0.362	− 0.05	0.14	− 0.02	0.630	− 0.12	0.07
PR mother negative parenting	0.03	0.572	− 0.07	0.12	0.00	0.953	− 0.10	0.09
PR father positive parenting	0.01	0.844	− 0.09	0.11	− 0.03	0.541	− 0.13	0.07
PR father negative parenting	0.01	0.854	− 0.09	0.11	0.02	0.668	− 0.08	0.12
Sex twins^a^	− 0.03	0.595	− 0.12	0.07	0.00	0.974	− 0.10	0.10
SES	0.01	0.927	− 0.09	0.10	0.01	0.874	− 0.09	0.10
C17 (N = 489)								
CR mother positive parenting	− 0.14	**0.003**	− 0.22	− 0.05	− 0.15	**0.001**	− 0.23	− 0.06
CR mother negative parenting	0.04	0.420	− 0.05	0.13	0.13	**0.004**	0.04	0.22
CR father positive parenting	− 0.02	0.742	− 0.10	0.07	− 0.03	0.505	− 0.12	0.06
CR father negative parenting	0.17	**0.000**	0.08	0.25	0.16	**0.000**	0.07	0.25
PR mother positive parenting	0.01	0.887	− 0.08	0.10	− 0.05	0.269	− 0.14	0.04
PR mother negative parenting	0.08	0.071	− 0.01	0.17	0.15	**0.001**	0.06	0.23
PR father positive parenting	0.02	0.682	− 0.07	0.11	− 0.01	0.839	− 0.10	0.08
PR father negative parenting	0.04	0.435	− 0.05	0.12	− 0.02	0.723	− 0.11	0.07
Sex twins^a^	− 0.01	0.761	− 0.10	0.08	0.08	0.100	− 0.01	0.16
SES	− 0.02	0.680	− 0.11	0.07	0.08	0.086	− 0.01	0.17

Bold indicates *p* < .05

*INT* internalizing, *EXT* externalizing, *C* cohort, *CI* 95% confidence interval, *PR* parental report, *CR* child report, *SES* socio-economic status

^a^1 = male, 2 = female

In the next step, we ran multiple regression analyses including all perceived differences in parenting behavior as well as their interactions with SES as predictors for MZ differences in problem behavior separately for every cohort to test whether the univariate associations remain significant after controlling for the others. The full models were then tested against nested models, in which all interaction terms were set to zero. Except for the C17 EXT model, dropping all SES interactions did not significantly worsen the model fit. For EXT in C17, we tested an additional model against the full model in which the only significant SES*parenting interaction was included, and all other interaction terms were set to zero. This model fitted the data as well as the full model. Estimates of the full models and all model comparisons can be found in Supplement 7, the final regression models are shown in Tables 4 and 5.

**Table 4:** Multiple regression coefficients to predict twin differences in Internalizing from twin differences in parenting

	Estimate	*SE*	*z*	*p(z)*	*CI − *	CI +	*β*
Model C05 (*N* = 426, *R*^*2*^ = 0.03)							
CR mother positive parenting	0.06	0.05	1.38	0.167	− 0.03	0.15	0.10
CR mother negative parenting	− 0.02	0.04	− 0.54	0.589	− 0.11	0.06	− 0.05
CR father positive parenting	0.02	0.05	0.43	0.669	− 0.08	0.12	0.03
CR father negative parenting	0.00	0.05	− 0.09	0.932	− 0.09	0.09	− 0.01
PR mother positive parenting	0.07	0.05	1.22	0.221	− 0.04	0.17	0.07
PR mother negative parenting	0.03	0.05	0.68	0.495	− 0.06	0.12	0.04
PR father positive parenting	0.06	0.06	1.02	0.307	− 0.06	0.18	0.06
PR father negative parenting	0.01	0.06	0.18	0.860	− 0.11	0.13	0.01
Model C11 (*N* = 412, *R*^*2*^ = 0.06)							
**CR mother positive parenting**	**− 0.07**	**0.03**	**− 2.32**	**0.020**	**− 0.13**	**− 0.01**	**− 0.15**
CR mother negative parenting	0.06	0.03	1.94	0.052	0.00	0.13	0.13
CR father positive parenting	0.03	0.03	1.01	0.313	− 0.03	0.09	0.07
CR father negative parenting	0.04	0.03	1.40	0.161	− 0.02	0.10	0.10
PR mother positive parenting	0.07	0.07	0.89	0.372	− 0.08	0.21	0.05
PR mother negative parenting	0.02	0.07	0.28	0.776	− 0.11	0.15	0.01
PR father positive parenting	0.02	0.10	0.23	0.816	− 0.18	0.22	0.02
PR father negative parenting	0.02	0.08	0.26	0.792	− 0.13	0.17	0.02
Model C17 (*N* = 489, *R*^*2*^ = 0.06)							
**CR mother positive parenting**	**− 0.08**	**0.03**	**− 2.97**	**0.003**	**− 0.13**	**− 0.03**	**− 0.16**
CR mother negative parenting	− 0.04	0.03	− 1.42	0.156	− 0.10	0.02	− 0.08
CR father positive parenting	0.02	0.02	0.74	0.462	− 0.03	0.06	0.04
**CR father negative parenting**	**0.11**	**0.03**	**3.59**	**0.000**	**0.05**	**0.17**	**0.21**
PR mother positive parenting	0.01	0.06	0.23	0.817	− 0.10	0.13	0.01
PR mother negative parenting	0.08	0.06	1.28	0.200	− 0.04	0.19	0.06
PR father positive parenting	0.05	0.08	0.70	0.484	− 0.09	0.20	0.04
PR father negative parenting	0.04	0.08	0.53	0.598	− 0.12	0.21	0.03

Significant estimates in **bold**

C cohort, CR child report, PR parental report, SE standard error, CI 95% confidence interval, β standardized estimate

**Table 5:** Multiple regression coefficients to predict twin differences in Externalizing from twin differences in parenting

	Estimate	*SE*	*z*	*p(z)*	CI −	CI +	*β*
Model C05 (*N* = 426, *R*^*2*^ = 0.06)							
CR mother positive parenting	0.07	0.06	1.03	0.302	− 0.06	0.19	0.08
CR mother negative parenting	0.04	0.06	0.65	0.518	− 0.08	0.16	0.06
CR father positive parenting	− 0.02	0.07	− 0.20	0.843	− 0.16	0.13	− 0.02
CR father negative parenting	− 0.07	0.07	− 1.03	0.304	− 0.20	0.06	− 0.12
**PR mother positive parenting**	**− 0.18**	**0.07**	**− 2.58**	**0.010**	**− 0.32**	**− 0.04**	**− 0.14**
PR mother negative parenting	0.09	0.06	1.59	0.112	− 0.02	0.20	0.08
PR father positive parenting	0.06	0.08	0.78	0.434	− 0.09	0.21	0.05
PR father negative parenting	0.11	0.08	1.33	0.185	− 0.05	0.26	0.08
Model C11 (*N* = 412, *R*^*2*^ = 0.07)							
CR mother positive parenting	− 0.05	0.03	− 1.55	0.120	− 0.11	0.01	− 0.09
CR mother negative parenting	0.03	0.03	0.88	0.377	− 0.04	0.09	0.06
CR father positive parenting	− 0.04	0.03	− 1.31	0.190	− 0.10	0.02	− 0.08
**CR father negative parenting**	**0.08**	**0.03**	**2.40**	**0.016**	**0.01**	**0.14**	**0.16**
PR mother positive parenting	− 0.03	0.08	− 0.35	0.729	− 0.18	0.13	− 0.02
PR mother negative parenting	− 0.03	0.07	− 0.48	0.632	− 0.17	0.11	− 0.03
PR father positive parenting	− 0.02	0.10	− 0.20	0.845	− 0.22	0.18	− 0.01
PR father negative parenting	0.04	0.08	0.53	0.593	− 0.11	0.19	0.03
Model C17 (*N* = 489, *R*^*2*^ = 0.09)							
**CR mother positive parenting**	**− 0.08**	**0.02**	**− 3.17**	**0.002**	**− 0.12**	**− 0.03**	**− 0.16**
CR mother negative parenting	0.03	0.03	1.04	0.300	− 0.02	0.08	0.06
CR father positive parenting	0.01	0.02	0.37	0.709	− 0.03	0.05	0.02
**CR father negative parenting**	**0.06**	**0.03**	**2.26**	**0.024**	**0.01**	**0.12**	**0.13**
PR mother positive parenting	− 0.05	0.05	− 0.83	0.409	− 0.15	0.06	− 0.04
**PR mother negative parenting**	**0.13**	**0.05**	**2.44**	**0.015**	**0.03**	**0.24**	**0.12**
PR father positive parenting	0.03	0.07	0.39	0.696	− 0.11	0.16	0.02
PR father negative parenting	− 0.04	0.08	− 0.48	0.634	− 0.19	0.11	− 0.03
SES*CR father negative parenting	**0.08**	**0.03**	**2.83**	**0.005**	**0.03**	**0.14**	**0.13**

Significant estimates in **bold**

C cohort, CR child report, PR parental report, *SES* socio-economic status, SE standard error, CI 95% confidence interval, β standardized estimate

#### Regression twin differences INT

In C05, after controlling for the other parenting differences, the associations between child-reported maternal positive parenting and parent-reported paternal positive parenting with INT did not reach significance anymore. This suggests that the significant findings in the correlation analyses were artefacts, and that no relationship between differences in parenting and differences in INT can be found. In C11, differences in parenting behavior explained 6% of the variance in INT differences. However, negative parenting could no longer contribute significantly to the prediction, leaving differences in child-reported maternal positive parenting as the only significant predictor. Finally, in the oldest cohort the multiple regression analyses replicates the univariate correlation pattern: 6% of the variance in INT differences could be mainly explained by significant differences in child-reported maternal positive parenting and child-reported paternal negative parenting.

#### Regression twin differences EXT

Of the four significant correlations in C05, only the association between differences in parent-reported maternal positive parenting and EXT differences remain significant in the regression model, which explains 6% of the variance. Similarly, in C11 about 7% of the variance were explained by differences in parenting, but of the four significant univariate correlations between parenting differences and differences in EXT, only differences in child-reported paternal negative parenting predict EXT differences. In contrast, for the oldest cohort three of four significant correlations remain significant in the regression model. Of these, merely differences in child-reported maternal negative parenting could not explain a relevant part of the variance in EXT differences anymore. Additionally, the interaction between SES and differences in child reported paternal negative parenting was found to contribute significantly to the variance proportion of 9% explained by differences in parenting behavior, pointing to a cumulative effect of paternal negative parenting and SES such as if children from families with higher SES perceived more differences in negative paternal parenting, also more differences in EXT symptoms are reported compared to children from lower SES families.

## Discussion

The aim of the current study was to investigate the unique environmental contribution of maternal and paternal positive and negative parenting to the development of problem behavior in different age cohorts in a representative German sample. Therefore, we used the twin difference method, which provides a rigorous test of true non-shared environmentally mediated effects, because it controls for genetic effects and shared environmental experiences. Our results show that particularly child-reported less positive maternal and more negative paternal parenting contribute significantly to the non-shared environmental variance of problem behavior. Although we did not find a clear pattern across age groups, structural differences in the associations between parenting differences and differences in problem behavior were found.

Our findings from the correlation analyses largely supported our hypotheses: We found that differences in negative parenting positively predict MZ differences in problem behavior, and differences in positive parenting negatively predict MZ differences in problem behavior. The only exceptions were for the youngest cohort, but since these correlations disappeared in the multiple regression, it is very likely that they are artefacts, so we refrain from interpreting them. Also, between-rater associations were generally weaker than within-rater associations and often disappeared completely after controlling for genetic confounding, and we could show that, overall, paternal parenting has a similar association with problem behavior as maternal parenting if tested univariately.

In the multiple regression models, with on average 5% for INT and 7% for EXT, the percentage of the non-shared environmental variance in problem behavior explained by parenting is at the upper end of the expected range for such a strictly genetically controlled design, especially when examining intra-familial environmental experiences (Vitaro et al. 2009). This underlines how important individual parenting is regarding variation in children’s problem behavior, independent of genetic or shared environmental influences. Considering that non-shared environmental variance in classic twin studies is a combination of all individual experiences as well as measurement error, it becomes clear that the contribution of parenting we found in our study is substantial. Of course, since we only obtained self-reports, it is possible that the objective parenting behavior does not differ at all, i.e., that it would be per definition an influence of the shared environment. But even if this were the case, we were able to show that it can indeed act as a non-shared environmental source if it is *perceived* as different and that this perception is significantly related to problem behavior.

For INT, the unique environmental contribution of perceived parenting appears to be significant only from early adolescence onwards, whereas for younger children the association between INT and perceived parenting is entirely due to genetic effects, rGE and shared environment. This may indicate that interventions which focus on parenting behavior toward younger children aiming at the prevention of problem behavior might better target the overall family context rather than individual parenting behavior specifically. For the other cohorts, perceived parenting was consistently an important source of non-shared environment, with children's perceptions being considerably more relevant than parenting reported by parents. In early adolescence, child-reported positive parenting of the mother as primary caregiver appears to be more important than the father’s parenting, such as less perceived maternal positive parenting was associated with more INT symptoms. In later adolescence, additionally, adolescents who felt also more negatively treated by their father reported significantly more INT symptoms.

For EXT, there were several significant correlations between differences in parenting behavior and EXT differences in the youngest cohort, but multiple regression analyses revealed, that only differences in parent-reported maternal parenting contribute significantly to the variance of EXT differences. That is, the mother reported more EXT symptoms for the child for whom she also rated her parenting behavior as less positive. Although previous research found a bidirectional relationship (Pinquart 2017), we cannot determine the causal direction of the association in our cross-sectional study, but since the phenotypic relationship between EXT and positive parenting is not as pronounced as between EXT and negative parenting, it is reasonable to assume that it is the mother who reacts with less positive parenting or perceive their parenting as less positive when they are confronted with EXT symptoms rather than children showing more EXT symptoms due to less positive parenting (cf. Huh et al. 2006). Although in early adolescence children's perceived positive and negative parenting of both parents was correlated with EXT, in the multiple regression analysis only child-reported paternal negative parenting made a significant contribution to the variance of EXT differences. In later adolescence, the child who reports more EXT also perceived paternal parenting more negatively. This shows the importance of a differentiated view on maternal and paternal parenting in these age groups and emphasizes that interventions might need to focus more strongly on the relationship between father and child. Nevertheless, the child who perceived maternal parenting less positively, also reported more EXT symptoms, and the only cross-rater association in the twin differences design concerned parent-reported more maternal negative parenting, which was associated with child-reported more EXT symptoms. This shows that in later adolescence, maternal as well as paternal parenting are important contributors to non-shared environmental variance in EXT, but that for each parent, different aspects are crucial. Finally, family SES could have an influence on the association between child-reported paternal negative parenting and EXT: The negative effect of paternal negative parenting seems additionally increase in higher SES families. Since we have only included a simple interaction term in our model, we cannot make any further statement about the nature or direction of this interaction. It would be very interesting to investigate this further, as it was the only significant interaction we found.

When interpreting the results, we also have to keep in mind that problem behavior in the youngest cohort was assessed via parent reports, and in the older two cohorts via child reports. However, when comparing the MZ correlations for problem behavior in C05 with those in the other cohorts, we find that the parent-reported MZ correlations in C05 are barely higher than those recorded via child reports in the other cohorts, indicating that parental ratings may only marginally overestimate the twins’ similarity, if at all. Moreover, using child reports to assess problem behavior in older children and adolescents offers several advantages over parent reports: Prior research has shown that correlations between different raters are only moderate for problem behavior and decrease with children’s age (Achenbach et al. 1987; Hartley et al. 2011; Los Reyes and Kazdin 2005). This is mainly because INT and EXT symptoms of children can also be observed very differently in different situations (e. g., at home, in school). Additionally, parent–child agreement is also significantly higher for observable symptoms than for unobservable ones, independent of different situations (Comer and Kendall 2004). Furthermore, children in nonclinical samples seem to answer questions about their problem behavior honestly (Thornberry and Krohn 2000) and report even more undesirable behavior than their parents (Martin et al. 2002; Rescorla et al. 2013; Scourfield et al. 2004; Seiffge-Krenke and Kollmar 1998). Univariate genetic research has also shown that non-shared environmental influences play a greater role in self-reported problem behavior than in other-reported problem behavior (Burt 2009; Cheesman et al. 2017; Eaves et al. 1997; Scourfield et al. 2004). In this respect, it can be assumed that the assessment of child reports is likely to lead to a realistic assessment of problem behavior and provides a good basis for studies on the non-shared environment in particular.

Taken together, we found significant associations between perceived parenting and problem behavior after controlling for genetic confounding and were thus able to show that parenting may be a direct source of the non-shared environment. The differences between phenotypic correlations and the twin differences design confirm again that relations found in phenotypic analyses are highly genetically confounded and underline the importance of genetically controlled designs in the study of environmental effects.

However, the disappearance of most associations between parent-reported parenting and problem behavior may not only be explained by the control of genetic effects. In line with previous research, in our study parents rated their parenting behavior towards both children much more similarly than the children did themselves leading to a variance restriction in the twin differences design and thus to lower correlations. This mainly concerns parent-reported maternal negative parenting, which was clearly related to problem behavior in all cohorts at the phenotypic level, but no longer after controlling for genetic effects. Therefore, we cannot rule out that parent-reported parenting in our study underestimates the true relations. Considering also that observational studies tend to back up the children's rather than the parental perception on parenting (Knopik et al. 2017), it could be very useful to assess parenting from the child's perspective instead of relying on parents' self-reports in research and diagnostics to get a more realistic view. Additionally, in the phenotypic correlation analyses, negative parenting shows a significantly higher association with EXT than positive parenting in the youngest cohort, and partly in the older ones, while this difference is no longer significant in the twin differences design (Supplement 7). However, Hanisch et al. (2014) found in their randomized controlled trial of the Prevention Program for Externalizing Behavior (PEP) that reducing negative parenting proved to be the most important component of the intervention. Our findings therefore suggest that the greater importance of negative parenting for EXT may be due to shared environmental pathways rather than non-shared environmental pathways.

Associations of perceived parenting with problem behavior did not show systematic age-related trends in C05 to C17, indicating that the unique environmental contribution parenting makes to the variance of problem behavior is independent of increasing child autonomy and continuous age-related changes in parenting and problem behavior. This also supports the findings of longitudinal genetic research that non-shared environmental influences on problem behavior may be rather short-termed over the course of development and still differ noticeably for different age groups (Knopik et al. 2017). We also found no strong pattern with respect to differences between maternal and paternal parenting, although the results of the multiple regressions models suggest structural differences in different age groups.

### Strengths and limitations

Strengths of our study are the genetically informed design which reduces the risk of inflated associations between parenting and problem behavior, the integration of different informants and age groups, and the large sample size. Additionally, the representativeness of our sample allows us to assume that the results are transferable to the general population. However, this implies that we cannot make specific statements about populations with clinically relevant manifestations of INT and EXT. Apart from that, there are a few more limitations: First, the effect size of the non-shared environment seems to be substantially greater for extreme parenting-discordant and problem behavior-discordant twins than for twins who are more similar to each other in both parenting and problem behavior (Asbury et al. 2003; Burt et al. 2006), which would increase the associations between parenting and problem behavior for this subgroups, too. Second, fewer fathers than mothers were willing to participate in our study. Since we found significant differences in child-reported paternal positive and negative parenting between families with participating vs. non-participating fathers (Supplement 2), it should be noted that the non-significant results concerning parent-reported paternal parenting should be interpreted with caution, as selection effects may play a role in the sense that reports of participating fathers may not represent the full range of parenting but are biased towards more positive functioning (Costigan and Cox 2001). Therefore, we cannot rule out the possibility that associations between parent-reported paternal parenting and problem behavior were underestimated. Similarly, the associations between parent-reported paternal parenting and SES may also be underestimated, although we do not expect this to be decisive for our results. Third, we controlled for genetic and shared environmental influences, but not for other non-shared environmental influences. Thus, other unknown non-shared environmental influences (e. g., experiences with peers) could potentially be responsible for the found correlations, and differential parental treatment might be due to behavioral differences of the twins that arose under the influence of those confounders. Fourth, given the fact that differential parenting explains a relatively large, but in absolute terms rather smaller part of the variance in MZ differences, the current results suggest that most of the non-shared environmental variance in behavior problems is not due to differential parenting. This raises the question what other factors might account for non-shared environmental influences on behavior problems which should be answered in further studies. Finally, the cross-sectional design of this current study did not grant testing for causal effects between differences in parenting and differences in problem behavior. In addition to the possibility that parenting differences cause differences in behavioral problems there are at least two plausible causal links: (a) more problematic behavior of one twin may evoke more negative parenting and (b) more INT as well as more EXT symptoms might result in more negative perceptions of parenting. Further longitudinal and genetically informative research is needed to allow conclusions about the causal direction of the associations and to which extent passive, evocative, or both types of rGE are present.

## Conclusion

Our study underlines the necessity of controlling for genetic confounding to uncover the truly environmentally mediated (and thus environmentally influenceable) pathways between parenting and problem behavior. In summary, our results illustrate that both perceived maternal and paternal parenting behavior are systematically related to problem behavior across different age groups after controlling for genetic and shared environmental influences, thus providing a starting point for studying their causal role (e.g., in controlled experiments) and then incorporating them in interventions to reduce problem behavior. A main finding was that it is crucial to especially consider the children's perception of their parents’ interactions with them as it is more strongly related to the expression of problem behavior than the parental reports. Our differentiated view makes it possible to derive concrete recommendations based on children's perceptions. For example, we showed that paternal parenting is no less important than maternal parenting during adolescence, and that the father should therefore be involved in interventions to reduce problem behavior, even if he is not the main caregiver in traditional family models.

## Supplementary Information

Below is the link to the electronic supplementary material.

Supplementary file1 (PDF 137 KB)

Supplementary file2 (PDF 138 KB)

Supplementary file3 (PDF 104 KB)

Supplementary file4 (PDF 138 KB)

Supplementary file5 (PDF 134 KB)

Supplementary file6 (PDF 113 KB)

Supplementary file7 (PDF 204 KB)

Supplementary file8 (PDF 117 KB)

## Data Availability

The datasets generated by the survey research during and/or analyzed during the current study are available in the GESIS repository, https://doi.org/10.4232/1.13658.
