# Supplementary material for: Differences in Parenting Behavior are Systematic Sources of the Non-shared Environment for Internalizing and Externalizing Problem Behavior
Source: Behav Genet. 2022 Nov 3;53(1):25–39. doi: 10.1007/s10519-022-10125-8 (PMC9823082; doi:10.1007/s10519-022-10125-8)
Supplement: Supplementary file 1 — Supplementary file1 (PDF 137 KB) [file 10519_2022_10125_MOESM1_ESM.pdf]

Supplement 1. *Descriptive statistics.*

	C05[a]			C11			C17		
	N	Min/Max	M (SD)	N	Min/Max	M (SD)	N	Min/Max	M (SD)
Age Twins	426	4 / 6	5.00 (0.36)	412	10 / 12	11.00 (0.37)	489	16 / 18	17.00 (0.36)
Age Mother	424	24 / 54	36.35 (5.15)	410	28 / 54	42.19 (5.02)	472	34 / 61	47.24 (4.73)
Age Father	379	24 / 64	39.54 (6.26)	346	29 / 78	46.13 (5.84)	379	36 / 69	49.91 (5.01)
T1 INT	426	0 / 1.50	0.24 (0.25)	412	0 / 1.30	0.46 (0.29)	489	0 / 1.70	0.51 (0.33)
T2 INT	426	0 / 1.40	0.24 (0.24)	412	0 / 1.80	0.46 (0.32)	489	0 / 1.80	0.49 (0.30)
T1 EXT	426	0 / 1.70	0.52 (0.31)	412	0 / 1.50	0.55 (0.30)	489	0 / 1.50	0.43 (0.29)
T2 EXT	426	0 / 1.70	0.51 (0.30)	412	0 / 1.50	0.56 (0.31)	489	0 / 1.50	0.43 (0.28)
T1 CR Mother Positive Parenting	227	1 / 3	2.61 (0.43)	399	1.75 / 5	3.85 (0.57)	473	1 / 5	3.64 (0.76)
T2 CR Mother Positive Parenting	245	1 / 3	2.59 (0.43)	404	1.75 / 5	3.85 (0.63)	478	1 / 5	3.67 (0.76)
T1 CR Mother Negative Parenting	218	1 / 3	1.80 (0.43)	399	1 / 4.83	2.27 (0.63)	473	1 / 5	2.47 (0.70)
T2 CR Mother Negative Parenting	235	1 / 3	1.84 (0.41)	404	1 / 4.33	2.25 (0.62)	477	1 / 5	2.46 (0.68)
T1 PR Mother Positive Parenting	407	2.50 / 5	4.33 (0.48)	382	2.67 / 5	4.26 (0.52)	444	2.25 / 5	4.01 (0.58)
T2 PR Mother Positive Parenting	408	2.38 / 5	4.35 (0.49)	387	2.25 / 5	4.28 (0.53)	443	2.38 / 5	4.01 (0.60)
T1 PR Mother Negative Parenting	407	1.11 / 4.33	2.61 (0.51)	382	1 / 4.39	2.52 (0.57)	445	1.11 / 4.17	2.37 (0.59)
T2 PR Mother Negative Parenting	408	1.11 / 4.39	2.60 (0.52)	387	1 / 4.33	2.54 (0.58)	442	1 / 4.61	2.33 (0.64)
T1 CR Father Positive Parenting	196	1 / 3	2.50 (0.45)	373	1 / 5	3.44 (0.77)	427	1 / 5	3.03 (0.89)
T2 CR Father Positive Parenting	208	1 / 3	2.45 (0.46)	375	1 / 5	3.42 (0.83)	431	1 / 5	3.10 (0.90)
T1 CR Father Negative Parenting	187	1 / 3	1.81 (0.44)	373	1 / 4.67	2.21 (0.70)	427	1 / 4.06	2.32 (0.70)
T2 CR Father Negative Parenting	203	1 / 3	1.82 (0.47)	375	1 / 4.50	2.15 (0.67)	431	1 / 5	2.33 (0.68)
T1 PR Father Positive Parenting	299	2.38 / 5	3.94 (0.53)	265	2 / 5	3.84 (0.57)	296	2 / 5	3.56 (0.62)
T2 PR Father Positive Parenting	305	2.25 / 5	3.92 (0.54)	263	1.63 / 5	3.85 (0.61)	299	1.50 / 5	3.54 (0.63)
T1 PR Father Negative Parenting	299	1.22 / 4	2.51 (0.49)	265	1.28 / 4.06	2.45 (0.51)	296	1 / 3.94	2.24 (0.55)
T2 PR Father Negative Parenting	305	1 / 4.39	2.50 (0.53)	263	1.22 / 4.33	2.43 (0.55)	300	1 / 4.17	2.25 (0.58)
Family SES	368	12 / 89	58.69 (20.43)	357	14 /89	55.02 (21.89)	427	14 / 89	54.60 (21.35)

C, Cohort; T1, twin 1; T2, twin2; INT, internalizing; EXT, externalizing; [a] for C05, INT & EXT were assessed via parental report; PR, parental report; CR; child report; SES, socioeconomic status; N = complete data
