# Supplementary material for: Differences in Parenting Behavior are Systematic Sources of the Non-shared Environment for Internalizing and Externalizing Problem Behavior
Source: Behav Genet. 2022 Nov 3;53(1):25–39. doi: 10.1007/s10519-022-10125-8 (PMC9823082; doi:10.1007/s10519-022-10125-8)
Supplement: Supplementary file 2 — Supplementary file2 (PDF 138 KB) [file 10519_2022_10125_MOESM2_ESM.pdf]

Supplement 2 – Household Structure and Missing Analyses

Household Structure

Table 1S2. *Household structure.*

	C05		C11		C17		all cohorts	
	N	%	N	%	N	%	N	%
Both twins and both parents in the same household	371	87.1	329	79.9	328	67.1	1028	77.5
Both twins and mother in the same household, no information on father	51	12	75	18.2	130	26.6	256	19.3
Both twins and father in the same household, no information on mother	2	0.5	2	0.5	19	3.9	23	1.7
Both twins and mother in the same household, father in different household	2	0.5	5	1.2	7	1.4	14	1.1
Both twins and father in the same household, mother in different household	0	0	1	0.2	1	0.2	2	0.2
One twin and mother in same household, other twin and father in another household	0	0	0	0	2	0.4	2	0.2
Twins in different households, one parent in same household with one twin	0	0	0	0	2	0.4	2	0.2
Total	426	100	412	100	489	100	1327	100

C, cohort.

Missing Analyses of Participating Fathers

Since only 8% of mothers but 36% of fathers did not provide information on their parenting behavior, we examined whether the rate of participating fathers was the same across cohorts and whether twin pairs whose fathers did not participate differed on all relevant variables from those with participating fathers. Table 2S2 shows frequencies of paternal participation for each cohort, Table 3S2 shows all significant group differences between families with participating and non-participating fathers.

Table 2S2. *Participating fathers * twin birth cohort.*

	C05		C11		C17		total	
	N	%	N	%	N	%	N	%
Not participating	127[a]	29.8	149[a. b]	36.2	198[b]	40.5	474	35.7
Participating	299[a]	70.2	263[a. b]	63.8	291[b]	59.5	853	64.3
Total	426	100	412	100	489	100	1327	100

χ^2 (3) = 11.36, p = .003; C, cohort; [a, b], each superscript letter indicates a subset of twin birth cohort categories whose column shares are not significantly different from each other at the .05 level.

Table 3S2. *T-tests participating vs. non-participating fathers (significant variables only).*

	N (fp. / fnp.)	*M (SD)* fp.	*M (SD)* fnp.	*F*	*p (F)*	*T*	*df*	*p (T)*
**C05**								
CR Father Negative Parenting	158 / 36	1.87 (0.44)	1.68 (0.43)	0.099	.753	2.389	192	.018
SES	286 / 82	60.11 (20.45)	53.77 (19.67)	0.980	.323	2.494	366	.013
**C11**								
CR Father Positive Parenting	252 / 120	3.57 (0.72)	3.19 (0.88)	4.121	.043	4.077	197.112	<.001
SES	253 / 104	58.17 (21.54)	47.36 (20.89)	0.407	.524	4.349	355	<.001
**C17**								
EXT	291 / 198	0.40 (0.27)	0.47 (0.30)	3.818	.051	-2.858	487	.004
CR Father Positive Parenting	286 / 140	3.17 (0.84)	2.91 (0.97)	4.623	.032	2.663	242.744	.008
CR Father Negative Parenting	286 / 140	2.37 (0.66)	2.19 (0.72)	1.899	.169	2.627	424	.009
SES	277 / 150	58.15 (19.79)	48.03 (22.61)	7.304	.007	4.609	272.902	<.001

fp., father participating; fnp., father not participating; C, cohort; CR, child report; EXT, externalizing; SES, socio-economic status.
