# Supplementary material for: Differences in Parenting Behavior are Systematic Sources of the Non-shared Environment for Internalizing and Externalizing Problem Behavior
Source: Behav Genet. 2022 Nov 3;53(1):25–39. doi: 10.1007/s10519-022-10125-8 (PMC9823082; doi:10.1007/s10519-022-10125-8)
Supplement: Supplementary file 3 — Supplementary file3 (PDF 104 KB) [file 10519_2022_10125_MOESM3_ESM.pdf]

**Supplement 3.** *Reliabilities (Cronbach's α).*

| | C05<br>T1 / T2 | C11<br>T1 / T2 | C17<br>T1 / T2 | All C<br>both twins |
|---|---|---|---|---|
| CR/PR INT[a] | .68 / .68 | .64 / .70 | .71 / .67 | .68 |
| CR/PR EXT[a] | .74 / .73 | .65 / .68 | .69 / .68 | .70 |
| CR Mother Positive Parenting | .65 / .67 | .60 / .69 | .80 / .80 | .70 |
| CR Mother Negative Parenting | .72 / .71 | .66 / .64 | .71 / .68 | .68 |
| PR Mother Positive Parenting | .73 / .78 | .76 / .79 | .80 / .83 | .78 |
| PR Mother Negative Parenting | .64 / .70 | .74 / .77 | .73 / .79 | .73 |
| CR Father Positive Parenting | .73 / .71 | .81 / .83 | .86 / .87 | .80 |
| CR Father Negative Parenting | .73 / .77 | .74 / .73 | .73 / .73 | .74 |
| PR Father Positive Parenting | .74 / .78 | .77 / .84 | .78 / .83 | .79 |
| PR Father Negative Parenting | .62 / .69 | .67 / .73 | .72 / .76 | .70 |

C, cohort; T1, twin 1; T2, twin2; INT, internalizing; EXT, externalizing; PR, parental report; CR; child report; [a] for C05, INT & EXT were assessed via parental report.
