# Supplementary material for: Differences in Parenting Behavior are Systematic Sources of the Non-shared Environment for Internalizing and Externalizing Problem Behavior
Source: Behav Genet. 2022 Nov 3;53(1):25–39. doi: 10.1007/s10519-022-10125-8 (PMC9823082; doi:10.1007/s10519-022-10125-8)
Supplement: Supplementary file 4 — Supplementary file4 (PDF 138 KB) [file 10519_2022_10125_MOESM4_ESM.pdf]

Supplement 4 – Exploratory Factor Analyses Parenting

Child-Reported Parenting

Table 1S4. *Factor analysis child-reported parenting mother, cohort 05.*

Factor name	Scale	Factor loadings 1	2
1. Negative Parenting	CR negative communication mother	**.859**	-.153
	CR psychological control mother	**.794**	
	CR inconsistent parenting mother	**.632**	.130
2. Positive Parenting	CR monitoring mother		**.899**
	CR warmth mother		**.890**

Factor loadings > .10 (Promax), CR, child report; **bold**, highest loadings.

Table 2S4. *Explained variance child-reported parenting mother, cohort 05.*

component	eigenvalue	% of variance	cumulative %
1	1.927	38.546	38.546
2	1.494	29.889	68.435
3	0.754	15.072	83.507
4	0.466	9.323	92.830
5	0.358	7.170	100

Table 3S4. *Factor analysis child-reported parenting father, cohort 05.*

Factor name	Scale	Factor loadings 1	2
1. Negative Parenting	CR negative communication father	**.856**	
	CR psychological control father	**.852**	
	CR inconsistent parenting father	**.617**	.190
2. Positive Parenting	CR monitoring father		**.867**
	CR warmth father		**.760**

Factor loadings > .10 (Promax), CR, child report; **bold**, highest loadings.

Table 4S4. *Explained variance child-reported parenting father, cohort 05.*

component	eigenvalue	% of variance	cumulative %
1	2.058	41.158	41.158
2	1.169	23.376	64.534
3	0.813	16.264	80.797
4	0.554	11.080	91.877
5	0.406	8.123	100

Table 5S4. *Factor analysis child-reported parenting mother, cohort 11 & 17.*

Factor name	Scale	Factor loadings 1	2
1. Negative Parenting	CR psychological control mother	**.787**	.290
	CR negative communication mother	**.786**	-.175
	CR inconsistent parenting mother	**.650**	
2. Positive Parenting	CR monitoring mother	.196	**.853**
	CR warmth mother	-.276	**.733**

Factor loadings > .10 (Promax), CR, child report; **bold**, highest loadings.

Table 6S4. *Explained variance child-reported parenting mother, cohort 11 & 17.*

component	eigenvalue	% of variance	cumulative %
1	1.918	38.351	38.351
2	1.246	24.920	63.271
3	0.734	14.678	77.949
4	0.661	13.223	91.171
5	0.441	8.829	100

Table 7S4. *Factor analysis child-reported parenting father, cohort 11 & 17.*

Factor name	Scale	Factor loadings 1	2
1. Negative Parenting	CR negative communication father	**.853**	-.189
	CR psychological control father	**.807**	.182
	CR inconsistent parenting father	**.592**	
2. Positive Parenting	CR warmth father		**.858**
	CR monitoring father	.111	**.833**

Factor loadings > .10 (Promax), CR, child report; **bold**, highest loadings.

Table 8S4. *Explained variance child-reported parenting father, cohort 11 & 17.*

component	eigenvalue	% of variance	cumulative %
1	1.750	35.007	35.007
2	1.498	29.957	64.964
3	0.824	16.484	81.448
4	0.565	11.307	92.755
5	0.362	7.245	100

Parent-Reported Parenting

Table 9S4. *Factor analysis parent-reported parenting mother, cohorts 05, 11 & 17.*

Factor name	Scale	Factor loadings 1	2
1. Negative Parenting	PR negative communication mother	**.793**	-.130
	PR psychological control mother	**.778**	.198
	PR inconsistent parenting mother	**.663**	
2. Positive Parenting	PR warmth mother		**.869**
	PR monitoring mother		**.846**

Factor loadings > .10 (Promax), PR, parent report; **bold**, highest loadings.

Table 10S4. *Explained variance parent-reported parenting mother, cohorts 05, 11 & 17.*

component	eigenvalue	% of variance	cumulative %
1	1.742	34.834	34.834
2	1.465	29.302	64.136
3	0.758	15.168	79.305
4	0.576	11.527	90.831
5	0.458	9.169	100

Table 11S4. *Factor analysis parent-reported parenting father, cohorts 05, 11 & 17.*

Factor name	Scale	Factor loadings 1	2
1. Negative Parenting	PR negative communication father	**.821**	-.118
	PR psychological control father	**.785**	.223
	PR inconsistent parenting father	**.606**	-.129
2. Positive Parenting	PR warmth father		**.845**
	PR monitoring father		**.824**

Factor loadings > .10 (Promax), PR, parent report; **bold**, highest loadings.

Table 12S4. *Explained variance parent-reported parenting father, cohorts 05, 11 & 17.*

component	eigenvalue	% of variance	cumulative %
1	1.694	33.876	33.876
2	1.438	28.765	62.641
3	0.804	16.071	78.712
4	0.615	12.307	91.019
5	0.449	8.981	100
