# Supplementary material for: Differences in Parenting Behavior are Systematic Sources of the Non-shared Environment for Internalizing and Externalizing Problem Behavior
Source: Behav Genet. 2022 Nov 3;53(1):25–39. doi: 10.1007/s10519-022-10125-8 (PMC9823082; doi:10.1007/s10519-022-10125-8)
Supplement: Supplementary file 5 — Supplementary file5 (PDF 134 KB) [file 10519_2022_10125_MOESM5_ESM.pdf]

Supplement 5 – Correlations Parenting

Table 1S5. *Correlations between child- and parent-reported parenting.*

	r	*p*	*lower 95% CI*	*upper 95% CI*
**C05** (N = 426)				
Mother Positive Parenting	.13	.006	.04	.23
Mother Negative Parenting	.30	.000	.21	.39
Father Positive Parenting	.18	.000	.08	.27
Father Negative Parenting	.06	.260	-.04	.15
**C11** (N = 412)				
Mother Positive Parenting	.25	.000	.16	.34
Mother Negative Parenting	.28	.000	.19	.36
Father Positive Parenting	.33	.000	.24	.42
Father Negative Parenting	.22	.000	.12	.31
**C17** (N = 489)				
Mother Positive Parenting	.38	.000	.30	.45
Mother Negative Parenting	.40	.000	.32	.47
Father Positive Parenting	.38	.000	.30	.46
Father Negative Parenting	.30	.000	.22	.38

C, cohort.

Table 2S5. *Correlations between mother- and father-reported parenting.*

	r	*p*	*lower 95% CI*	*upper 95% CI*
**C05** (N = 426)				
CR Positive Parenting	.62	.000	.56	.68
CR Negative Parenting	.62	.000	.56	.68
PR Positive Parenting	.18	.000	.09	.27
PR Negative Parenting	.32	.000	.23	.40
**C11** (N = 412)				
CR Positive Parenting	.51	.000	.44	.58
CR Negative Parenting	.68	.000	.62	.73
PR Positive Parenting	.30	.000	.21	.39
PR Negative Parenting	.33	.000	.25	.42
**C17** (N = 489)				
CR Positive Parenting	.48	.000	.40	.54
CR Negative Parenting	.52	.000	.45	.58
PR Positive Parenting	.39	.000	.31	.46
PR Negative Parenting	.29	.000	.20	.37

C, cohort; CR, child report; PR, parental report.
