# Supplementary material for: Differences in Parenting Behavior are Systematic Sources of the Non-shared Environment for Internalizing and Externalizing Problem Behavior
Source: Behav Genet. 2022 Nov 3;53(1):25–39. doi: 10.1007/s10519-022-10125-8 (PMC9823082; doi:10.1007/s10519-022-10125-8)
Supplement: Supplementary file 6 — Supplementary file6 (PDF 113 KB) [file 10519_2022_10125_MOESM6_ESM.pdf]

Supplement 6. *Fisher's z-values (Fisher, 1925) representing differences in the magnitude of correlations in cohort 11 vs. cohort 17.*

	z	*p*
Correlations between twin differences in INT and twin differences in…		
CR Mother Positive Parenting	0.454	.650
CR Mother Negative Parenting	**2.253**	**.024**
CR Father Positive Parenting	-0.179	.858
CR Father Negative Parenting	0.092	.927
PR Mother Positive Parenting	0.582	.561
PR Mother Negative Parenting	-0.807	.419
PR Father Positive Parenting	-0.134	.893
PR Father Negative Parenting	-0.388	.698
Correlations between twin differences in EXT and twin differences in…		
CR Mother Positive Parenting	0.076	.939
CR Mother Negative Parenting	0.472	.637
CR Father Positive Parenting	-1.775	.076
CR Father Negative Parenting	0.554	.579
PR Mother Positive Parenting	0.388	.698
PR Mother Negative Parenting	**-2.236**	**.025**
PR Father Positive Parenting	-0.313	.754
PR Father Negative Parenting	0.551	.581

INT, internalizing; EXT, externalizing; CR, child report; PR, parental report; all z-values are two-tailed.

References

Fisher, R.A. (1925). *Statistical Methods for Research Workers.* Oliver and Boyd, Edinburgh, Scotland.
