# Supplementary material for: Differences in Parenting Behavior are Systematic Sources of the Non-shared Environment for Internalizing and Externalizing Problem Behavior
Source: Behav Genet. 2022 Nov 3;53(1):25–39. doi: 10.1007/s10519-022-10125-8 (PMC9823082; doi:10.1007/s10519-022-10125-8)
Supplement: Supplementary file 7 — Supplementary file7 (PDF 204 KB) [file 10519_2022_10125_MOESM7_ESM.pdf]

Supplement 7

– Multiple Regressions with SES Interactions and Model Comparsions

Table 1S1. *Multiple regression coefficients to predict twin differences in Internalizing and Externalizing from twin differences in parenting with SES interactions.*

	Estimate	*SE*	*z*	*p(z)*	CI -	CI +	*β*
**INT**							
Model C05 (N = 426, R^2 = .05)							
CR Mother Positive Parenting	.07	.05	1.38	.167	-.03	.17	.11
CR Mother Negative Parenting	-.02	.05	-0.44	.663	-.11	.07	-.04
CR Father Positive Parenting	.02	.06	0.39	.698	-.09	.14	.04
CR Father Negative Parenting	.01	.06	0.12	.907	-.10	.12	.01
PR Mother Positive Parenting	.03	.06	0.51	.613	-.09	.16	.03
PR Mother Negative Parenting	.04	.05	0.84	.403	-.06	.14	.05
PR Father Positive Parenting	.06	.06	0.94	.348	-.07	.19	.06
PR Father Negative Parenting	.00	.07	0.06	.953	-.12	.13	.00
SES*CR Mother Positive Parenting	-.06	.08	-0.76	.450	-.21	.09	-.07
SES*CR Mother Negative Parenting	-.03	.07	-0.37	.713	-.17	.11	-.04
SES*CR Father Positive Parenting	-.03	.08	-0.40	.686	-.19	.13	-.05
SES*CR Father Negative Parenting	-.02	.07	-0.30	.763	-.17	.12	-.04
SES*PR Mother Positive Parenting	.11	.09	1.25	.212	-.06	.28	.10
SES*PR Mother Negative Parenting	.02	.06	0.35	.725	-.10	.15	.02
SES*PR Father Positive Parenting	-.08	.08	-1.09	.276	-.23	.07	-.08
SES*PR Father Negative Parenting	.10	.07	1.48	.140	-.03	.24	.09
Model C11 (N = 412, R^2 = .08)							
CR Mother Positive Parenting	-.08	.03	-2.54	.011	-.14	-.02	-.16
CR Mother Negative Parenting	.07	.03	2.20	.028	.01	.14	.15
CR Father Positive Parenting	.02	.03	0.71	.481	-.04	.08	.05
CR Father Negative Parenting	.04	.03	1.40	.161	-.02	.10	.10
PR Mother Positive Parenting	.04	.08	0.45	.654	-.12	.19	.02
PR Mother Negative Parenting	.03	.07	0.37	.711	-.11	.16	.02
PR Father Positive Parenting	.03	.11	0.31	.755	-.17	.24	.02
PR Father Negative Parenting	.02	.08	0.24	.814	-.13	.17	.02
SES*CR Mother Positive Parenting	-.03	.03	-0.79	.428	-.09	.04	-.05
SES*CR Mother Negative Parenting	.07	.04	2.05	.041	.00	.14	.14
SES*CR Father Positive Parenting	.03	.03	0.99	.325	-.03	.10	.07
SES*CR Father Negative Parenting	-.04	.03	-1.16	.247	-.11	.03	-.08
SES*PR Mother Positive Parenting	.11	.10	1.12	.261	-.08	.30	.06
SES*PR Mother Negative Parenting	.01	.08	0.08	.938	-.14	.15	.00
SES*PR Father Positive Parenting	-.12	.12	-1.01	.311	-.34	.11	-.07
SES*PR Father Negative Parenting	-.03	.09	-0.38	.704	-.21	.14	-.03

	Estimate	*SE*	*z*	*p(z)*	CI -	CI +	*β*
Model C17 (N = 489, R^2 = .08)							
CR Mother Positive Parenting	-.07	.03	-2.56	.011	-.12	-.02	-.14
CR Mother Negative Parenting	-.04	.03	-1.53	.127	-.10	.01	-.09
CR Father Positive Parenting	.01	.02	0.51	.610	-.03	.06	.03
CR Father Negative Parenting	.11	.03	3.69	.000	.05	.17	.22
PR Mother Positive Parenting	-.04	.06	-0.66	.511	-.17	.08	-.04
PR Mother Negative Parenting	.09	.06	1.43	.154	-.03	.21	.07
PR Father Positive Parenting	.07	.08	0.88	.377	-.08	.22	.06
PR Father Negative Parenting	.04	.08	0.52	.603	-.12	.21	.03
SES*CR Mother Positive Parenting	.04	.04	1.08	.282	-.03	.11	.06
SES*CR Mother Negative Parenting	-.04	.04	-0.97	.333	-.11	.04	-.06
SES*CR Father Positive Parenting	-.02	.03	-0.50	.615	-.07	.04	-.03
SES*CR Father Negative Parenting	.02	.04	0.54	.588	-.05	.10	.03
SES*PR Mother Positive Parenting	-.11	.08	-1.46	.146	-.26	.04	-.09
SES*PR Mother Negative Parenting	-.03	.07	-0.42	.673	-.17	.11	-.02
SES*PR Father Positive Parenting	-.02	.10	-0.18	.859	-.20	.17	-.01
SES*PR Father Negative Parenting	.07	.10	0.69	.493	-.13	.28	.04
**EXT**							
Model C05 (N = 426, R^2 = .12)							
CR Mother Positive Parenting	.09	.07	1.34	.182	-.04	.23	.12
CR Mother Negative Parenting	.08	.07	1.24	.216	-.05	.21	.12
CR Father Positive Parenting	-.01	.08	-0.06	.953	-.17	.16	-.01
CR Father Negative Parenting	-.11	.08	-1.40	.162	-.27	.05	-.19
PR Mother Positive Parenting	-.22	.09	-2.57	.010	-.39	-.05	-.17
PR Mother Negative Parenting	.07	.07	1.00	.318	-.06	.19	.06
PR Father Positive Parenting	.03	.08	0.32	.752	-.14	.19	.02
PR Father Negative Parenting	.11	.08	1.30	.193	-.06	.28	.09
SES*CR Mother Positive Parenting	-.17	.11	-1.57	.117	-.38	.04	-.17
SES*CR Mother Negative Parenting	-.15	.10	-1.47	.141	-.34	.05	-.16
SES*CR Father Positive Parenting	-.02	.12	-0.19	.848	-.26	.21	-.03
SES*CR Father Negative Parenting	.17	.11	1.61	.107	-.04	.37	.22
SES*PR Mother Positive Parenting	-.06	.12	-0.46	.647	-.29	.18	-.04
SES*PR Mother Negative Parenting	.01	.09	0.17	.867	-.16	.18	.01
SES*PR Father Positive Parenting	-.20	.10	-1.94	.052	-.39	.00	-.14
SES*PR Father Negative Parenting	.02	.09	0.26	.795	-.15	.20	.02
Model C11 (N = 412, R^2 = .09)							
CR Mother Positive Parenting	-.05	.03	-1.53	.125	-.11	.01	-.09
CR Mother Negative Parenting	.03	.03	0.92	.360	-.04	.10	.06
CR Father Positive Parenting	-.05	.03	-1.58	.113	-.11	.01	-.10
CR Father Negative Parenting	.08	.03	2.54	.011	.02	.15	.17
PR Mother Positive Parenting	-.09	.09	-1.01	.313	-.25	.08	-.06
PR Mother Negative Parenting	-.03	.07	-0.36	.720	-.17	.12	-.02

	Estimate	*SE*	*z*	*p(z)*	CI -	CI +	*β*
PR Father Positive Parenting	.01	.11	0.07	.941	-.20	.22	.01
PR Father Negative Parenting	.08	.08	0.98	.325	-.08	.23	.06
SES*CR Mother Positive Parenting	.03	.03	0.84	.400	-.04	.09	.05
SES*CR Mother Negative Parenting	.02	.04	0.58	.563	-.05	.09	.04
SES*CR Father Positive Parenting	.03	.04	0.75	.456	-.04	.10	.05
SES*CR Father Negative Parenting	-.05	.04	-1.46	.145	-.12	.02	-.10
SES*PR Mother Positive Parenting	.10	.11	0.98	.328	-.10	.31	.06
SES*PR Mother Negative Parenting	-.04	.08	-0.42	.676	-.20	.13	-.02
SES*PR Father Positive Parenting	-.18	.12	-1.54	.125	-.41	.05	-.10
SES*PR Father Negative Parenting	.01	.09	0.15	.884	-.17	.19	.01
Model C17 (N = 489, R^2 = .11)							
CR Mother Positive Parenting	-.08	.02	-3.30	.001	-.13	-.03	-.17
CR Mother Negative Parenting	.03	.03	1.02	.308	-.02	.08	.06
CR Father Positive Parenting	.01	.02	0.45	.656	-.03	.05	.02
CR Father Negative Parenting	.06	.03	2.34	.020	.01	.12	.13
PR Mother Positive Parenting	-.05	.06	-0.79	.427	-.16	.07	-.04
PR Mother Negative Parenting	.14	.06	2.49	.013	.03	.25	.12
PR Father Positive Parenting	.05	.07	0.74	.460	-.09	.19	.05
PR Father Negative Parenting	-.04	.08	-0.55	.583	-.19	.11	-.03
SES*CR Mother Positive Parenting	-.03	.03	-1.06	.289	-.09	.03	-.06
SES*CR Mother Negative Parenting	-.04	.03	-1.19	.233	-.11	.03	-.07
SES*CR Father Positive Parenting	-.01	.03	-0.33	.741	-.06	.04	-.02
SES*CR Father Negative Parenting	.10	.03	3.04	.002	.04	.17	.18
SES*PR Mother Positive Parenting	-.04	.07	-0.54	.587	-.18	.10	-.03
SES*PR Mother Negative Parenting	.05	.06	0.81	.421	-.07	.18	.04
SES*PR Father Positive Parenting	-.07	.09	-0.81	.416	-.24	.10	-.06
SES*PR Father Negative Parenting	.11	.09	1.11	.266	-.08	.29	.07

C, cohort; INT, internalizing; EXT, externalizing; CR, child report; PR, parental report; SES, socio-economic status; SE, standard error; CI, 95% confidence interval, β, standardized estimate.

Table 2S7. *Model comparisons.*

	df	*AIC*	*BIC*	χ^2	χ^2 *diff*	*df diff*	*p*
**INT**							
C05							
full model	0	1020.0	1709.3	0.00			
without SES interactions	8	1009.2	1666.0	51.54	51.54	8	.741
C11							
full model	0	5645.7	6329.2	0.00			
without SES interactions	8	5637.3	6288.7	76.79	76.79	8	.465
C17							
full model	0	6928.1	7640.8	0.00			
without SES interactions	8	6918.2	7597.3	60.52	60.52	8	.641
**EXT**							
C05							
full model	0	1201.7	1891.0	0.00			
without SES interactions	8	1199.1	1855.9	13.36	13.36	8	.100
C11							
full model	0	5698.0	6381.6	0.00			
without SES interactions	8	5689.8	6341.2	78.01	78.01	8	.453
C17a							
full model	0	6833.8	7546.5	0.00			
without SES interactions	8	6836.1	7515.2	18.33	18.33	8	.019
C17b							
full model	0	6833.8	7546.5	0.00			
with sig. SES interaction only	7	6829.0	7512.4	9.26	9.26	7	.234

INT, internalizing; EXT, externalizing; C, cohort; SES, socio-economic status.
