# Supplementary material for: Differences in Parenting Behavior are Systematic Sources of the Non-shared Environment for Internalizing and Externalizing Problem Behavior
Source: Behav Genet. 2022 Nov 3;53(1):25–39. doi: 10.1007/s10519-022-10125-8 (PMC9823082; doi:10.1007/s10519-022-10125-8)
Supplement: Supplementary file 8 — Supplementary file8 (PDF 117 KB) [file 10519_2022_10125_MOESM8_ESM.pdf]

Supplement 8. *Comparison of absolute correlation coefficients for correlations between positive parenting and externalizing vs. correlations between negative parenting and externalizing.*

	C05		C11		C17	
	z	*p*	*z*	*p*	*z*	*p*
Phenotypic						
CR M PP - CR M NP	**-3.523**	**.000**	-0.896	.185	-1.613	.053
CR F PP - CR F NP	**-2.986**	**.001**	-0.980	.163	**-1.761**	**.039**
PR M PP – PR M NP	**-2.461**	**.007**	**-1.729**	**.042**	**-2.836**	**.002**
PR F PP – PR F NP	-0.766	.222	-1.267	.103	-1.164	.122
Twin differences						
CR M PP - CR M NP	1.020	.846	-0.234	.407	0.318	.625
CR F PP - CR F NP	-0.937	.174	-0.722	.235	**-2.064**	**.020**
PR M PP – PR M NP	0.605	.727	0.300	.618	-1.512	.065
PR F PP – PR F NP	-1.053	.146	0.129	.551	-0.109	.457

C, Cohort; CR, child report; PR, parent report; M, mother; F, father; PP, positive parenting; NP, negative parenting; **bold**, p < .05.
Correlation coefficients were compared with Fisher's z (Fisher, 1925).

References

Fisher, R.A. (1925). *Statistical Methods for Research Workers.* Oliver and Boyd, Edinburgh, Scotland.
